# Delayed hypersensitivity as a pathophysiological mechanism in cutaneous lesions due to SARS-CoV-2

**DOI:** 10.11604/pamj.supp.2020.35.2.24980

**Published:** 2020-07-16

**Authors:** Mostafa Rafai, Jalal Elbenaye, Sana Sabry, Hicham Janah

**Affiliations:** 1 Department of physiology, faculty of medicine and pharmacy, Hassan II university, Casablanca, Morocco,; 2 Department of dermatology, Moulay Ismail military hospital, Meknes, Morocco,; 3 Faculty of medicine and pharmacy, Sidi Mohamed Ben Abdellah University, 30000, Fes, Morocco,; 4 Department of pneumology, Avicenne military hospital, Marrakech, Morocco

**Keywords:** COVID-19, chilblains, purpura, erythema multiforme, hypersensitivity

## To the Editors of the Pan African Medical Journal

**Figure 1:**
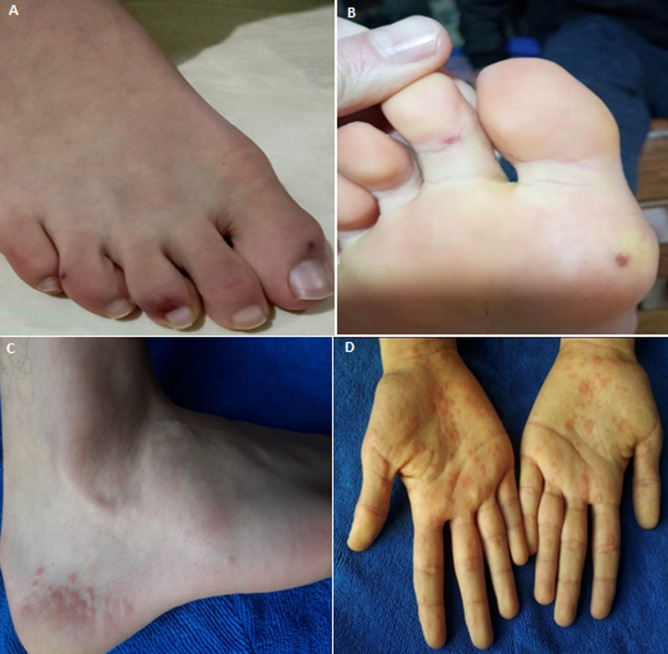
A) chilblains-like lesions on the toes; B) erythematopurpuric lesions of soles; C) erythematous maculopapular lesions of the heel; D) targetoid aspect on the palms

A 17-year-old adolescent with no medical history; documented to have a mild SARS-CoV-2 infection (clinical symptoms and minimal peripheral ground-glass opacities in both lungs in chest CT); had chilblains-like lesions on the toes (Figure 1 A) and asymptomatic erythematopurpuric lesions of soles (Figure 1 B) on the fourth day of the onset of COVID-19 symptoms. He took vitamin C only. There were no thrombocytopenia, no hypercoagulability except a slight increase of inflammatory markers. Sars-cov-2 RT-PCR was negative. On the fifteenth day of the onset of symptoms, he developed mild itching and painless erythematous maculopapular lesions of heels (Figure 1 C) with targetoid aspect on the palms (Figure 1 D). There was no mucosal involvement. No recent episode of recurrent herpes or drugs intake were noted. Reported COVID-19 associated cutaneous manifestations are various. Some occur early as exanthem, urticaria, chickenpox like rash; mainly affecting the trunk [1]; while others appear later like chilblains and maculopapular lesions with acral distribution [2]. This suppose that there would be two types of lesions according to two different pathophysiological mechanisms: first and early one which would be linked to viremia and a second; late; related to immunological and inflammatory response during the disease.

Our patient had presented chilblains-like lesions and acral purpura concomitantly, followed few days later by maculopapular lesions with targetoid lesions reminiscent of erythema multiforme. Same presentations were reported: 02 cases with chilblains-like lesions evolving to erythemato-papular targetoid lesions [3]; maculopapular lesions in heels [4]. All these observations were seen in healthy young patients, with negative SARS-CoV-2 RT-PCR, appear late and would have a good prognosis. These findings suggest that acral lesions would be the clinical expression of type III and/or IV hypersensitivity targeting the small vessels of skin then responsible for endothelial activation, dermal and perivascular lymphoid infiltrate. Histological observations corroborate this hypothesis [2-6]. These suggestions require more investigation by means of SARS-CoV-2 serological tests, more relevant histology with immunohistochemistry and immunofluorescence and finally a serum assay of complement and immunological factors.
